# Supplementary material for: Cancer Is Associated with Alterations in the Three-Dimensional Organization of the Genome
Source: Cancers (Basel). 2019 Nov 27;11(12):1886. doi: 10.3390/cancers11121886 (PMC6966451; doi:10.3390/cancers11121886)
Supplement: Supplementary file 1 [file cancers-11-01886-s001.zip › cancers-632042-suppl.-final/cancers-632042-supplementary Figures.docx]

Article

Cancer Is Associated with Alterations in the Three-Dimensional Organization of the Genome

Lifei Li ^1^, Nicolai K. H. Barth ^1^, Christian Pilarsky ^2^ and Leila Taher ^1,3,^*


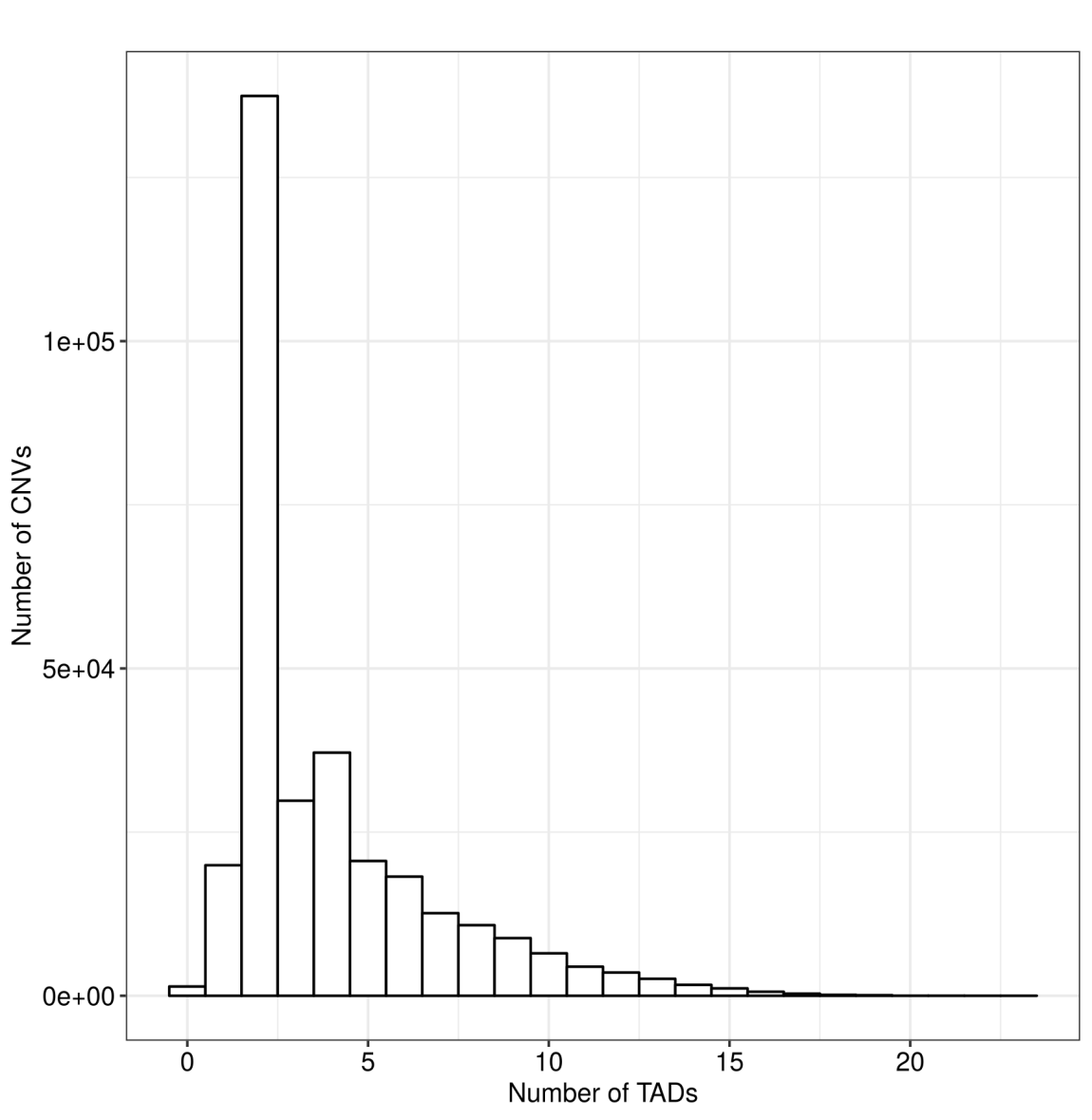


**Figure S1.** Number of TADs overlapping with each CNV.


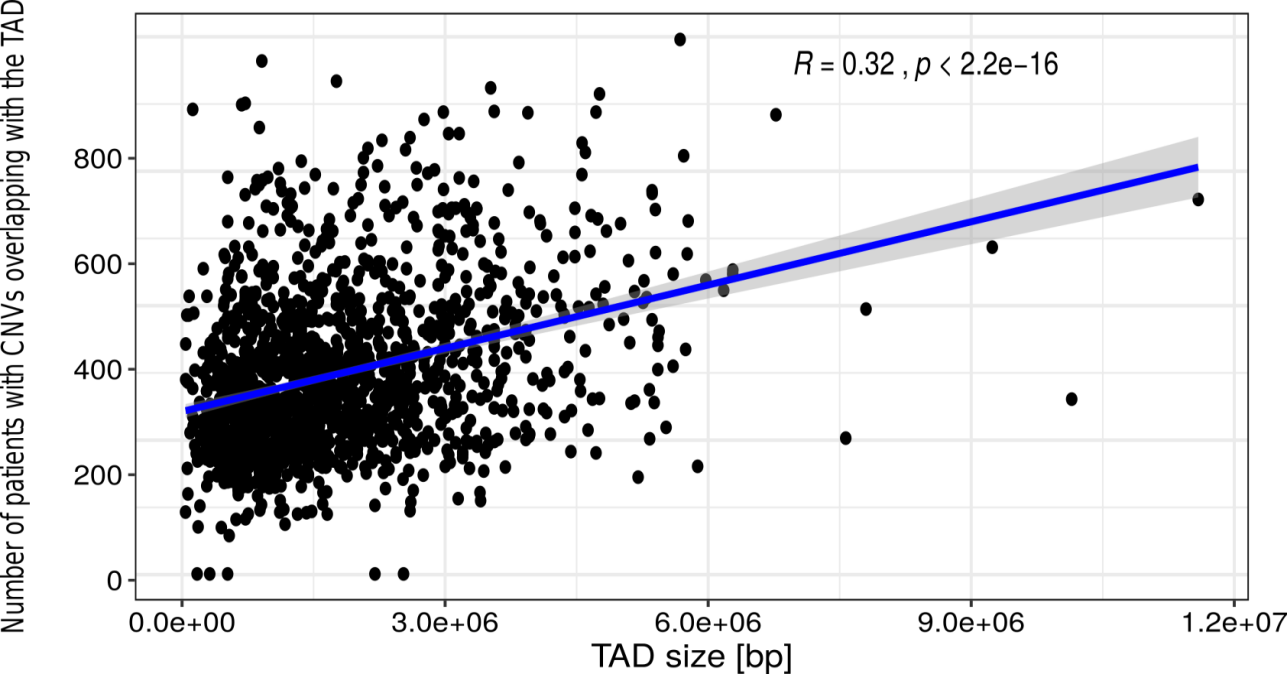


**Figure S2.** The size of the TADs and the number of CNVs overlapping with the TADs are only weakly correlated. Number of patients with one or more CNVs overlapping with a TAD as a function of the size of the TAD. The blue line is fitted linear model describing the relationship between TAD size and number of CNVs overlapping with the TAD. The grey shade represents the 95% confidence interval of the fitted value. R is the Spearman’s correlation coefficient and p is the P-value for Spearman’s correlation test.


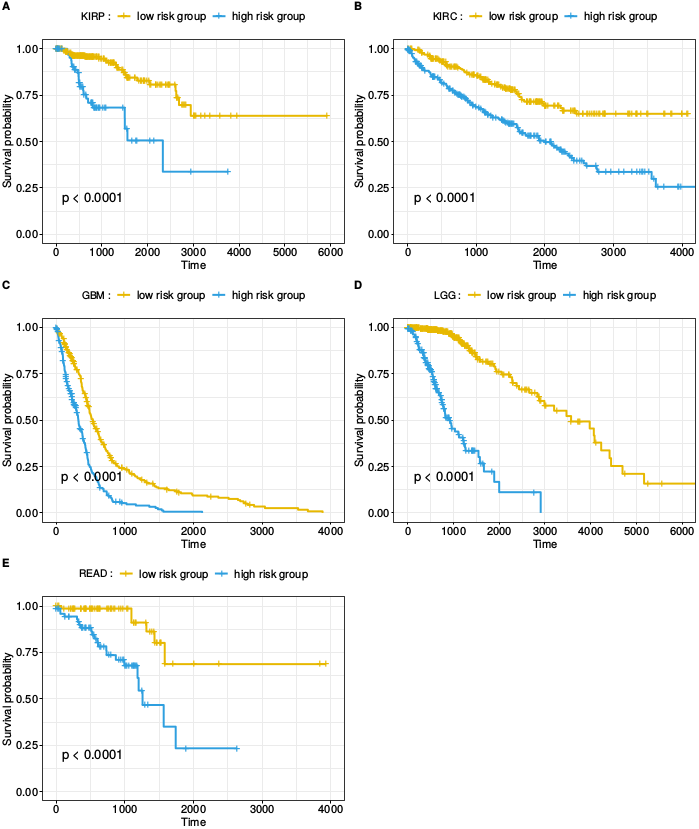


**Figure S3.** Kaplan-Meier analysis for KIRP, KIRC, GBM, LGG AND READ. Patients were separated into high- (blue) and a low-risk (yellow) groups according to the prognostic features of the final LASSO Cox regression model and subjected to Kaplan-Meier analysis. A) KIRP; B) KIRC; C) GBM; D) LGG; E) READ.


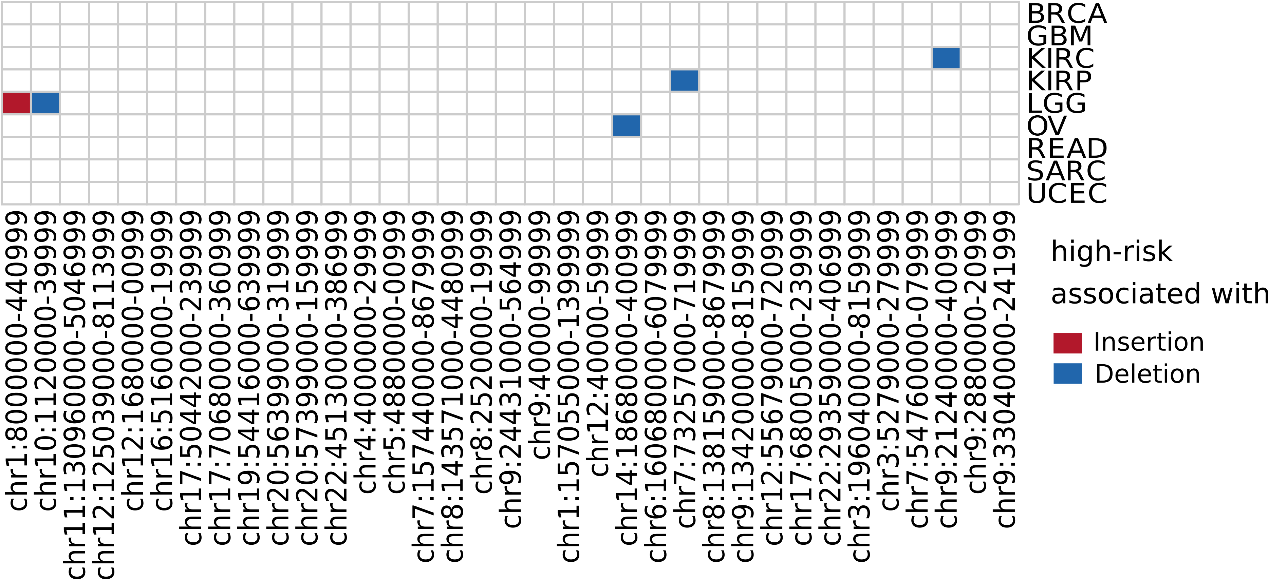


**Figure S4.** Five out of the 35 prognostic TADs displayed significant different survivals depending on whether patients had a deletion or an insertion. Red indicates TADs for which insertions in the TAD are associated with significantly lower patient survival (as compared to deletions); blue TADs for which deletions in the TAD are associated with significantly lower patient survival (as compared to insertions). The remaining prognostic TADs (featured in the figure) showed no differences.


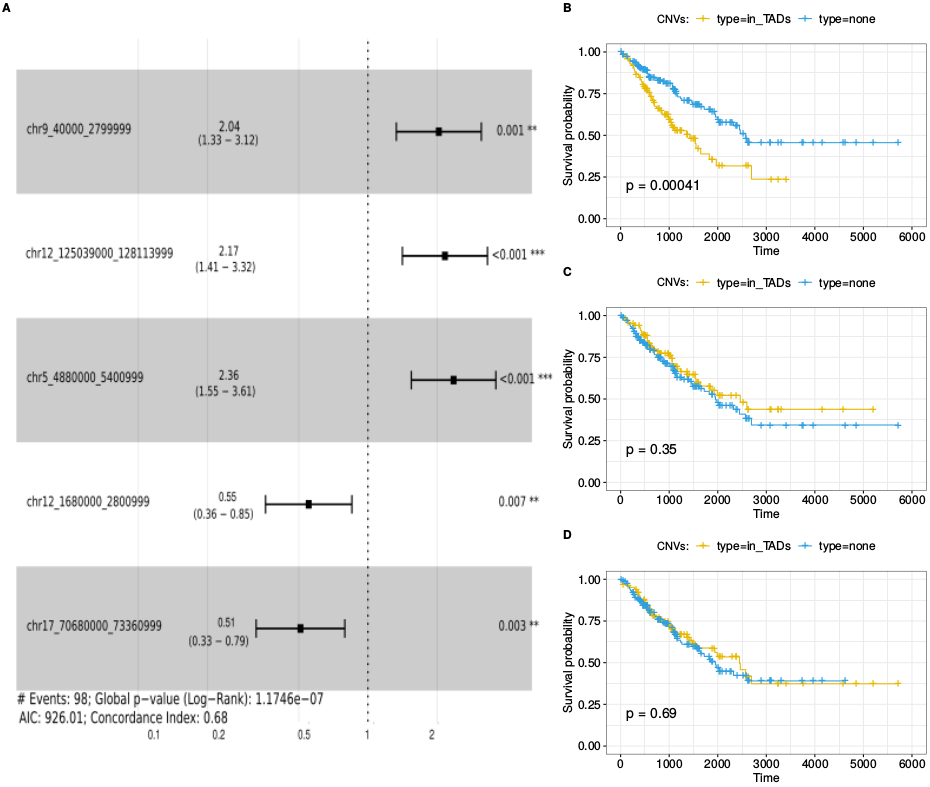


**Figure S5.** Survival analysis for SARC patients. (**A**) Hazard ratios with 95% confidence intervals for all prognostic (P-values < 0.05) TADs from the final LASSO Cox regression model. (**B–D**) Kaplan-Meier curves for patients separated according to the presence or absence of CNVs in prognostic TADs: (**B**) chr5:4880000-5400999; (**C**) chr12:1680000-2800999; and (**D**) chr17:70680000-73360999.


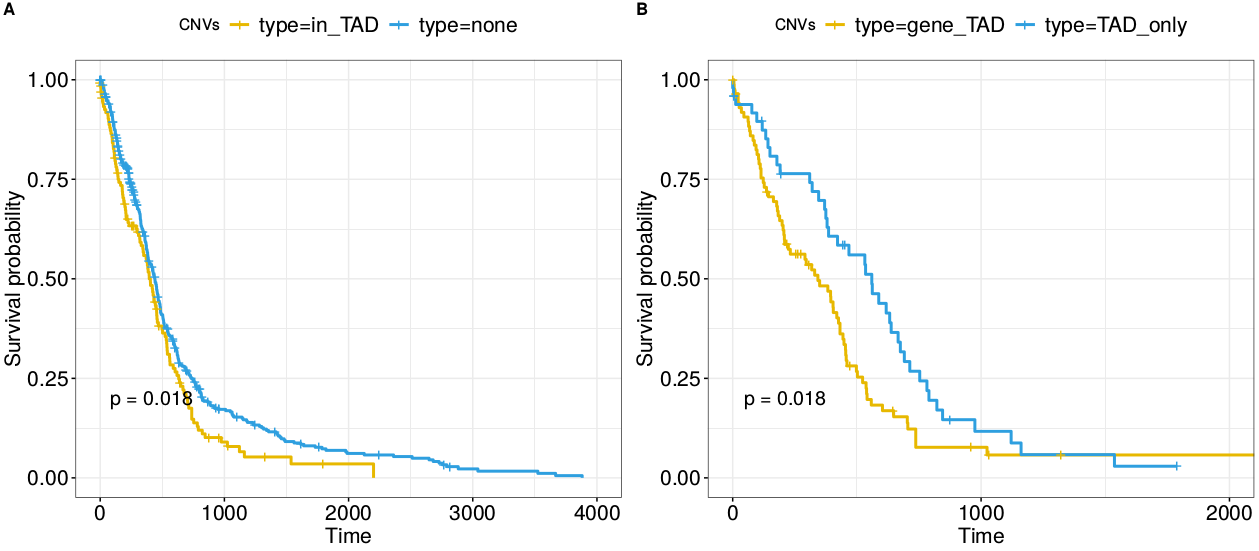


**Figure S6.** Kaplan-Meier survival analysis for GBM patients separated according to the presence or absence of CNVs in the prognostic TAD chr12:55679000-57720999. (**A**) Survival of the 136 patients that had CNVs in the TAD (yellow) compared to that of the 453 patients that did not (blue). (**B**) Survival of the 87 patients that had CNVs in *DDIT3* (yellow) compared to that of the 49 patients that had CNVs in the TAD but not in *DDIT3* (blue).


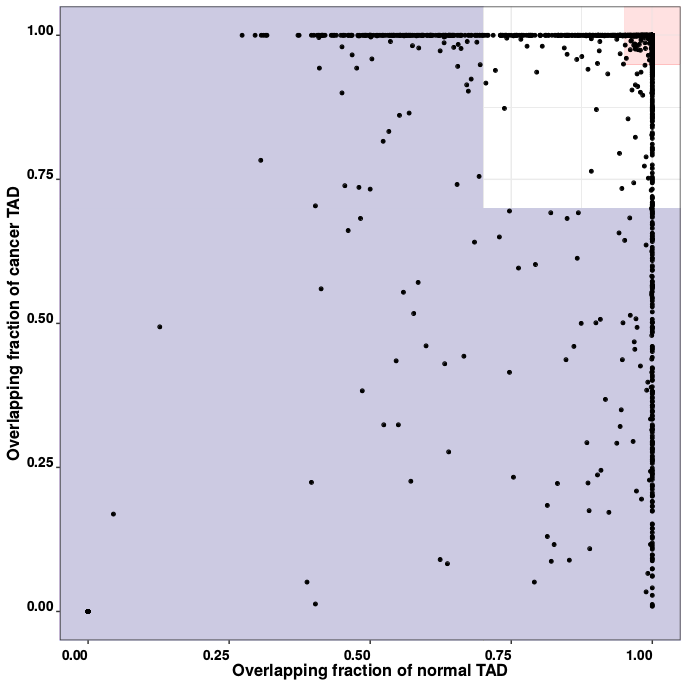


**Figure S7.** Fraction of mutual overlaps for normal and cancer TADs. Each dot represents a normal TAD. The x-axis shows the fraction of the sequence of the (normal) TAD that overlapped with a cancer TAD; if a (normal) TAD overlapped with multiple cancer TADs, we selected the TAD with the highest fraction. For the corresponding normal TAD, the y-axis shows the fraction of its sequence overlapping with the normal TAD. Constitutive TADs are highlighted in red; non-constitutive TADs are shown in purple; the remaining TADs were considered “ambiguous”.


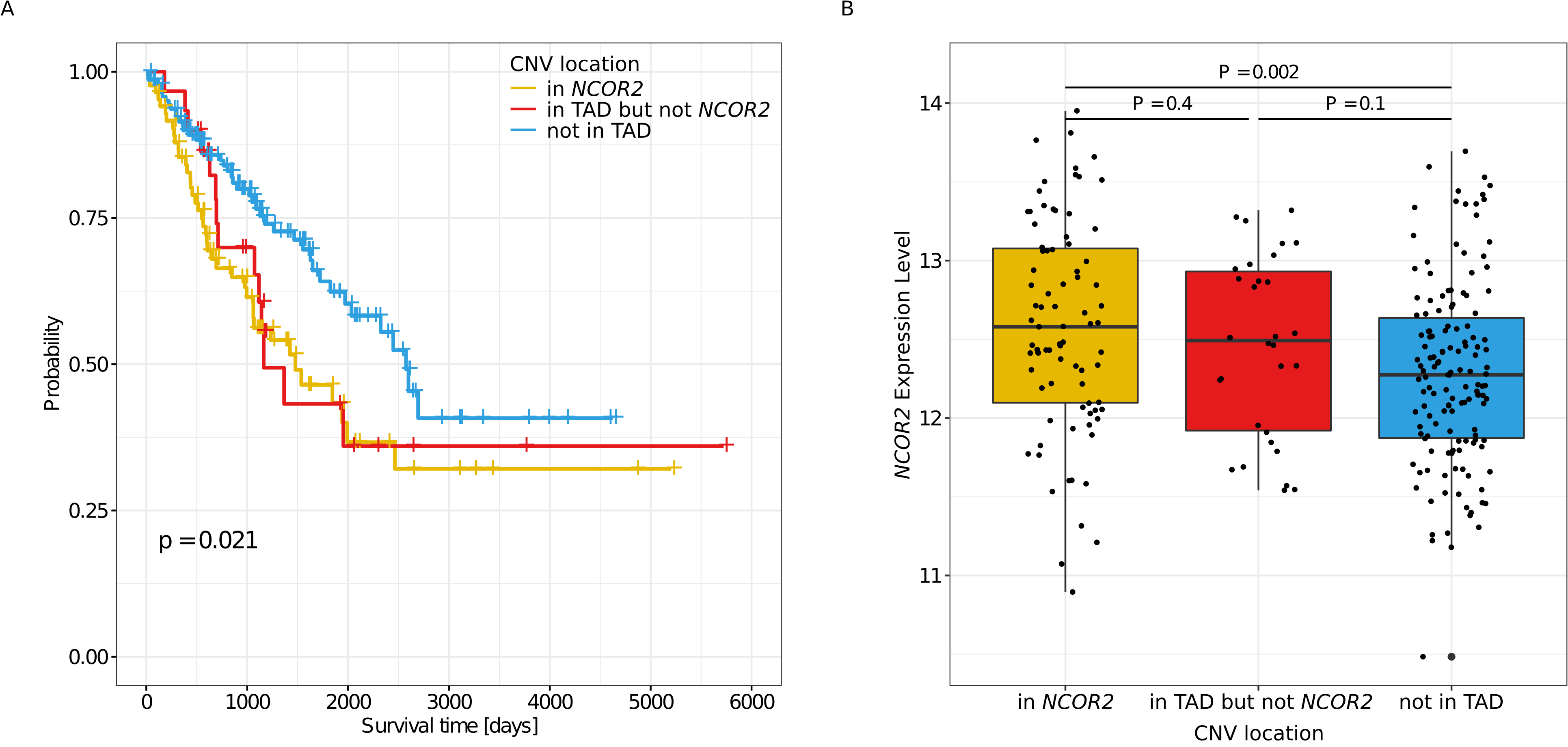


**Figure S8.** Kaplan-Meier survival analysis and *NCOR2* expression levels for SARC patients. Patients were separated into three groups: i) patients with CNVs in *NCOR2* (yellow); ii) patients with CNVs in the TAD chr12:125039000-128113999 but in *NCOR2* (red); and iii) patients with no CNVs in TAD chr12:125039000-128113999 (and, consequently, neither in *NCOR2*, blue): A) Kaplan-Meier survival analysis and B) expression level of *NCOR2*.


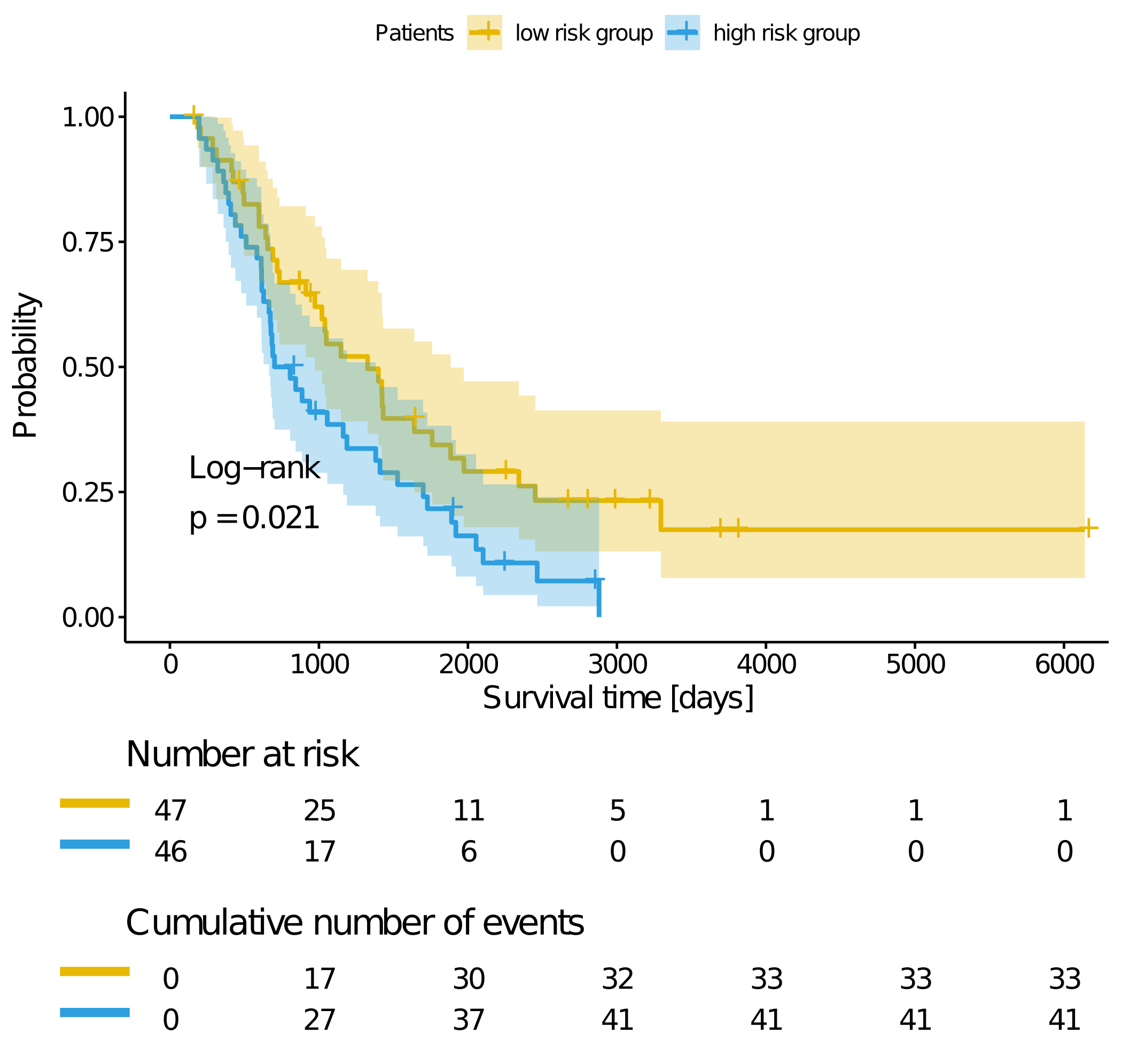


**Figure S9.** Kaplan-Meier survival analysis for “Ovarian cancer-AU” patients separated into high- (blue) and a low-risk (yellow) groups according to the prognostic features of the final TAD-based LASSO Cox regression model for OV.

**
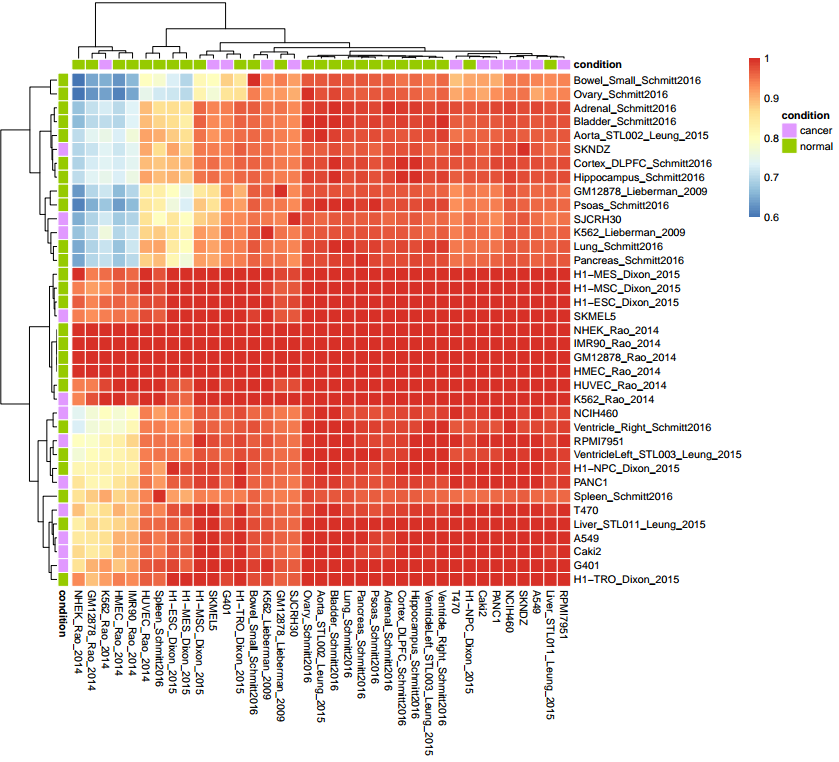
**

**Figure S10.** TAD maps of 24 normal tissues/cell lines and 11 cancer cell lines. The heatmap summarizes the pairwise similarities between every pair of samples (see the Materials and Methods section of the manuscript for details).


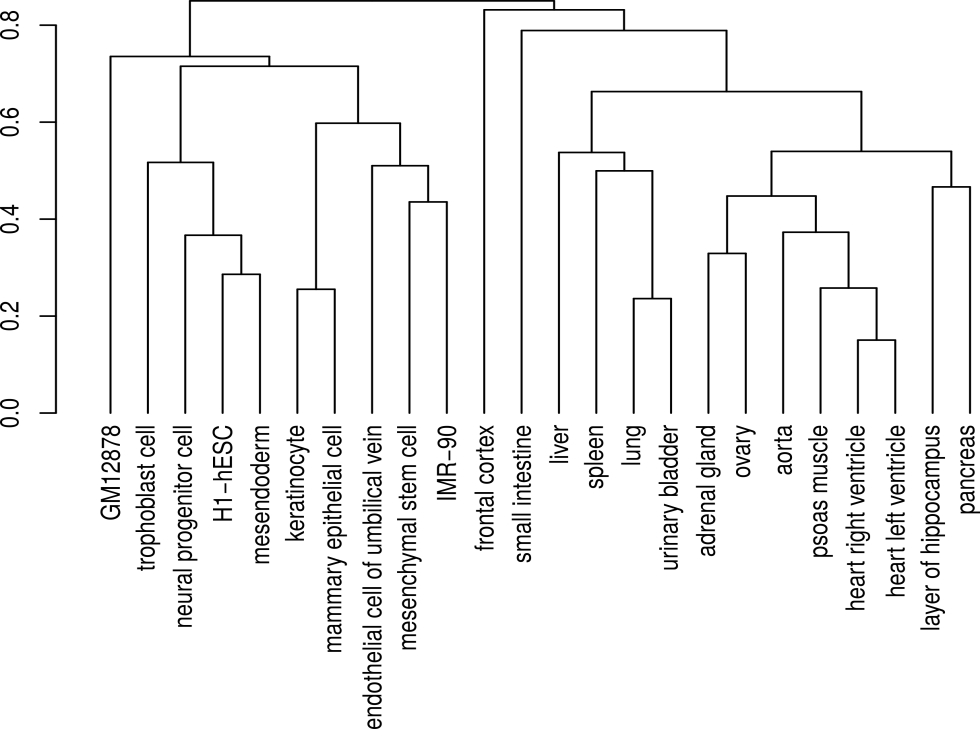


**Figure S11.** Relationship among 24 normal tissues, based on the similarity of their expression profiles.


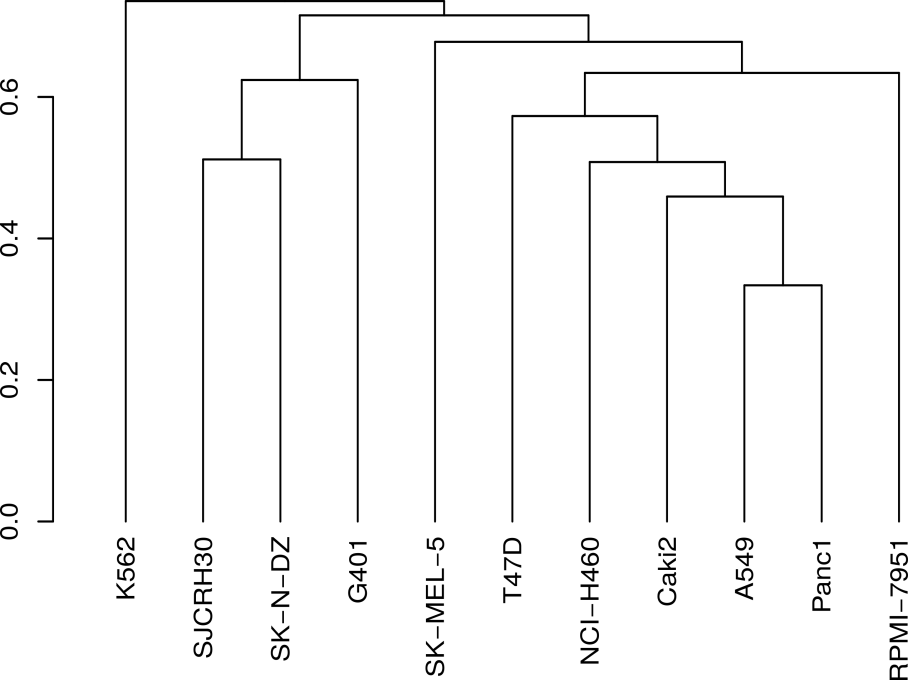


**Figure S12.** Relationship among eleven cancer cell lines, based on the similarity of their expression profiles.

| 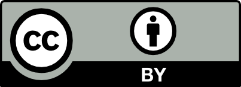 | © 2019 by the authors. Submitted for possible open access publication under the terms and conditions of the Creative Commons Attribution (CC BY) license (http://creativecommons.org/licenses/by/4.0/). |
| --- | --- |
